# QUANTIFYING PACE OF AGING AMONG OLDER ADULTS: AN ANALYSIS WITH HEALTH AND RETIREMENT STUDY

**DOI:** 10.1093/geroni/igae098.2246

**Published:** 2024-12-31

**Authors:** Arun Balachandran, Heming Pei, Meeraj Kothari, Benjamin Dominigue, Alex Furuya, Dan Belsky

**Affiliations:** Columbia University Irving Medical Center, New York, New York, United States; Columbia University, New York, New York, United States; Columbia University Irving Medical Center, New York, New York, United States; Stanford University, Stanford, California, United States; Columbia University Irving Medical Center, New York, New York, United States; Columbia University Irving Medical Center, New York, New York, United States

## Abstract

Rise in global population aging and age-related morbidities calls for measures that that can detect differences in aging trajectories in time to reduce and prevent disease burdens. Pace of Aging (PoA) measure captures longitudinal change in human physiology across multiple systems and captures within-individual changes. However, there has been no investigations of the measure around a sample of older adults to identify who are aging fast and who are most likely to gain from interventions. Using nine biomarkers representing three types of data (blood biomarkers, physical assessments, and functional tests) in the Health and Retirement Study (in 2006, 2008, 2010, 2012, 2014, and 2016), we studied PoA among 13,379 adults aged 40 or above at their first biomarker measurement. Our results extend analysis of PoA into later life and establish its associations with morbidity, disability, and mortality. Racial disparities in PoA represent opportunities for health intervention and prevention. Pace of Aging was more rapid in older as compared to younger participants. Independent of age, participants with faster Pace of Aging were at increased risk of cognitive impairment, incident chronic disease, disability, and death over follow-up compared to those with slower Pace of Aging (e.g. mortality HR=1.94 95%CI[1.82-2.08]). Population groups with shorter life expectancies exhibited faster Pace of Aging. Pace of Aging is a new tool for bio-social gerontology that can inform projections and evaluate trends in aging-related health changes in populations around the world.

